# The future of starch bioengineering: GM microorganisms or GM plants?

**DOI:** 10.3389/fpls.2015.00247

**Published:** 2015-04-23

**Authors:** Kim H. Hebelstrup, Domenico Sagnelli, Andreas Blennow

**Affiliations:** ^1^Department of Molecular Biology and Genetics, Aarhus University, Slagelse, Denmark; ^2^Department of Plant and Environmental Sciences, University of Copenhagen, Frederiksberg C, Denmark

**Keywords:** starch modification, biopharming, GM crops, starch phosphorylation, prebiotic, hybrid starch

## Abstract

Plant starches regularly require extensive modification to permit subsequent applications. Such processing is usually done by the use of chemical and/or physical treatments. The use of recombinant enzymes produced by large-scale fermentation of GM microorganisms is increasingly used in starch processing and modification, sometimes as an alternative to chemical or physical treatments. However, as a means to impart the modifications as early as possible in the starch production chain, similar recombinant enzymes may also be expressed *in planta* in the developing starch storage organ such as in roots, tubers and cereal grains to provide a GM crop as an alternative to the use of enzymes from GM microorganisms. We here discuss these techniques in relation to important structural features and modifications of starches such as: starch phosphorylation, starch hydrolysis, chain transfer/branching and novel concepts of hybrid starch-based polysaccharides. *In planta* starch bioengineering is generally challenged by yield penalties and inefficient production of the desired product. However, in some situations, GM crops for starch bioengineering without deleterious effects have been achieved.

## Introduction

Modified starches are important commodities used in many food and material applications. In 2011 the global annual production of pure native starch was 73 million tons and it is expected to reach 133.5 million tons in 2018 (http://www.strategyr.com/showsearchNew.asp). Raw starch is usually modified by industrial chemical and/or physical treatments. The most frequent chemical modifications are esterification, etherification or oxidation of hydroxyl groups. Physical treatments usually involve shaping and/or disruption by mechanical force or thermal treatments or a combination of both (Kaur et al., 2012). These starch modifications are made to change functionalities to meet demands for downstream uses. In addition to physical and chemical modifications, enzymes are used as additives in industrial processes and in food processing to modify starches. Starches are α-glucan polymers joined by α-1,4 linkages with additional branches of α-1,6 linkages. Organisms such as bacteria, fungi, plants, and animals contain genes for enzymes serving their demand for α-glucan digestion and/or biosynthesis (Ball et al., 2011; Cenci et al., 2013). Some of these genes can be used to generate genetically modified microorganisms, which can produce such enzymes on an industrial scale for starch modifications. Here we discuss a third scenario, where the plants themselves are used as bioreactors to generate the enzymes directly in the starch storage organs, so that the starch is already modified during its synthesis. The strategy may require that the plants have the capability to synthesize and translocate the enzymes to the subcellular compartments (amyloplasts), where starch is synthesized (Blennow et al., 2013). It may also require codon optimization of the gene encoding the enzyme and addition of plant specific N-terminal transit peptides, which directs the correct subcellular translocation. It is also a requirement that the enzymes are functional in the plant cell compartment environment, which may sometimes be very different from their natural environment with respect to parameters like temperature, pH and presence of co-factors. As an alternative, the enzymes may be engineered to meet the conditions in the plant cell compartments. However, optimal kinetic parameters of the enzymatic modifications may not always be necessary due to the very long exposure time and constant production of the new enzyme during crop storage organ development (weeks to months), as compared to industrial fermentation, where it is instrumental to have optimal physiological conditions in the processes in order to reduce production time (hours). In other situations it may actually be intentional that the enzymes are only activated post-harvest during subsequent processing of the crops. The latter is particularly useful in the case of hydrolytic enzymes as discussed below.

Amyloplasts in starch storage organs, in fact, represent natural bioreactors where an array of biosynthetic enzymes are orchestrated to build starch granules with a typical size of 1–100 μM (Blennow et al., 2013). Therefore in certain situations, starch bioengineering can be achieved by adjusting the activity of endogenous enzymes in crop storage organs, through endogenous gene overexpression, gene silencing or gene point mutations, without the need for insertion of foreign DNA into the crops. Such methods represent alternatives to transgenic GM crops and GM microorganisms in the form of so-called *cis*-genesis, TILLING and genome editing. In the *cis*-genic concept, only genome-endogenous DNA is inserted into a plant. The european food safety authority (EFSA) recently concluded that an equivalent level of hazard can be associated with *cis*-genesis as for conventional plant breeding, which should favor the *cis*-genic strategy as a non-GM technology (Schouten and Jacobsen, 2008; EFSA Panel on Genetically Modified Organisms, 2012). TILLING (Targeted Induced Local Lesions in Genomes) is a development of mutation breeding, where random mutations are induced by mutagenic compounds such as ethyl methanesulfonate (EMS) or sodium azide (NaN_3_) or by high-energy radiation. When combined with high-throughput molecular DNA screening techniques, targeted mutations in genes of interest can be identified (Chen et al., 2014). TILLING has recently been used to identify new alleles of starch-modifying and starch biosynthesis enzymes in cereals leading to new starch functionalities, such as increased amylose and resistant starch (Slade et al., 2012; Sparla et al., 2014). Genome editing is based on DNA modifying factors, which are orchestrated to recognize and induce small specific mutations such as single nucleotide substitutions and small (<20 bp) deletions/insertions. At present three main systems are available for plants: zinc-finger domain nucleases, TALEN factors and CRISPR. Zinc-fingers are DNA binding proteins with motifs derived from transcription factors. They can be engineered to bind to custom DNA binding motifs. When two such zinc-fingers are linked to nucleases that are activated upon dimerization, they permit specific recognition of flanking gene segments and the subsequent cleavage and mutagenesis of the adjacent DNA sequence (Desjarlais and Berg, 1992). Similarly, TALENs (Transcription activator-like nucleases) are DNA binding domains linked to nucleases (Boch et al., 2009; Cermak et al., 2011; Mahfouz et al., 2014). A third genome editing technology is the so-called CRISPR (clustered regularly interspaced short palindromic repeats) /Cas9/sgRNA system for targeted gene mutagenesis (Doudna and Charpentier, 2014; Mahfouz et al., 2014). Genome editing protocols are available for important starch crops, such as maize (*Zea mays*; Liang et al., 2014), bread wheat (*Triticum aestivum*) and rice (*Oryza sativa*; Shan et al., 2014), and the techniques have been used to engineer important agronomic traits, such as diseases resistances (Li et al., 2012; Wang et al., 2014). However, no genome editing based techniques have yet been reported to be used for starch bioengineering. At the present time, crops which undergo the above mentioned genome-editing techniques are regarded as GM according to European legislation. In addition, genetic transformation of crops is time- and labor intensive. For example the development time for a new transgenic cereal laboratory prototype line is typically 1.5–3 years.

## Starch Hydrolysis

Different hydrolytic strategies are currently used to produce mono- or disaccharide sugars from starch using enzymes. These processes typically include the use of α-amylases (EC 3.2.1.1), which hydrolyze α-1,4 linkages, producing maltodextrins, and β-amylase (EC 3.2.1.2) acting specifically on α-1,4 linkages from the non-reducing ends to produce β-maltose. Glucoamylases (EC 3.2.1.3) can be used to further hydrolyse β-limit dextrins, maltodextrins and maltose to glucose. Finally, hydrolases may be used in combination with isomerases to convert between isomeric forms of saccharides, such as in the case of glucose isomerase (EC 5.3.1.5) used to convert glucose into fructose (Kaur et al., 2012). Such hydrolytic processes can be moved from the processing tank directly into the plants by expression of hydrolytic enzymes in crop organs using a concept called “self-processing plants” (Santa-Maria et al., 2011). The concept relies on a “control switch” to activate post-harvest hydrolysis, so that preliminary hydrolysis during crop development is prevented. Typical approaches involve targeting of enzyme(s) to cellular sub-compartments different from the amyloplast, preventing direct physical contact with the developing starch granules, or the use of thermophilic or hyperthermophilic hydrolases, which remain inactive at ambient temperatures (<40°C) but that can be activated at temperatures above 60°C during industrial processing. Such strategies have been used with success in potato tuber (*Solanum tuberosum*, Beaujean et al., 2000), sweet potato (*Ipomoea batatas*, Santa-Maria et al., 2011), and japonica rice (*Oryza sativa* spp. *Japonica*, Xu et al., 2008). A life cycle assessment (LCA) of corn starch hydrolysis has indicated that replacement of 25% of the grain input with a self-processing corn that expresses such a transgenic thermostable α-amylase enzyme resulted in reduced production costs as well as an 11% reduction of total greenhouse gas emission, 7.7% reduction of water usage, 8.9% reduction in natural gas usage and a 5% increase in fermenter content capacity as compared to the use of externally added amylase (Urbanchuk et al., 2009).

## Branching and De-Branching Enzymes

Branching and de-branching enzymes represent two types of starch hydrolases that affect the number of α-1,6 linkages, and thereby the branching structure of starch. De-branching enzymes, such as pullulanase/limit dextrinase (EC 3.2.1.41) and isoamylase (EC 3.2.1.68), specifically hydrolyse α-1,6 linkages. Specific homologs of debranching enzymes are naturally involved in the biosynthesis of starch in plants. Their suppression results in production of so-called phytoglycogen (Fujita et al., 2003), which is a highly soluble and potentially prebiotic glucan resembling glycogen whilst their expression is associated with correct trimming of amylopectin across plant species (Utsumi et al., 2011; Streb and Zeeman, 2014).

Additional α-1,6 starch branching or formation of cyclic α-glucans can be generated through the use of α-glucanotransferases (van der Maarel and Leemhuis, 2013), such as branching enzyme (EC 2.4.1.18, Jensen et al., 2013), 4-α-glucanotransferases (EC 2.4.1.25), cyclodextrin glucanotransferases (EC 2.4.1.19), or 4,6-α-glucanotransferases (EC 2.4.1.X, Kralj et al., 2011). Both 4-α-glucanotransferases and cyclodextrin glucanotransferases are used extensively for the production of cyclodextrin and cycloamylose in fermentation. A cyclodextrin glucanotransferase from the bacterium *Klebsiella* has been expressed in potato tubers to generate cyclodextrins. However, the amount of cyclodextrin was ≤0.01% of the starch fraction (Oakes et al., 1991). A glycogen branching enzyme from *E. coli* has been used to increase the branching in potato tuber starch granules (Huang et al., 2013). Branching of starch in plants occurs naturally during starch biosynthesis catalyzed by endogenous glucanotransferases called starch branching enzymes (SBEs). Suppression of SBEs in most plants results in increased starch chain length as manifested by a combined increased of amylose content and amylopectin unit chain length (Regina et al., 2012). For example, the *amylose-extender* (ae) maize starch types contain above 70% amylose, which is caused by loss-of-function mutations in the SBEIIb gene (Stinard et al., 1993). Simultaneous suppression of all SBE genes in barley resulted in a starch type with >99% amylose (Carciofi et al., 2012b). In summary, approaches for expressing or modulating α-glucanotransferases directly in crops have focused on their activity during starch biosynthesis and crop development, which contrasts to the heat-activated starch hydrolase strategy mentioned above, where enzymes are activated only after crop harvest.

## Phosphorylation

Phosphorylation is the only known natural chemical modification of starch. The precise physiological function of natural starch phosphorylation is still discussed, even 100 years after its discovery (Fernbach, 1904; Samec, 1914). Natural highly phosphorylated starches, such as those synthesized in potato tubers and leaves, have a high hydration capacity and produce clear and very viscous pastes (Wiesenborn et al., 1994; Viksø-Nielsen et al., 2001). These functionalities can also be achieved through chemical phosphorylation. Natural starch phosphate is found as monoesters at both the C-3 and the C-6 positions of the glucose residues, where the C-6 phosphate represents approximately 70% of the total starch phosphate (Hizukuri et al., 1970; Tabata and Hizukuri, 1971; Bay-Smidt et al., 1994). The natural phosphate content is typically between 0.5 and 35 nmol phosphate esters per mg starch. Cereal storage starches generally have lower starch phosphate contents than tuber starches. For comparison, the levels that can be achieved through chemical esterification can be up to 0.4%_w/w_ P (≈130 nmol/mg starch) (Lim and Seib, 1993). Natural starch phosphorylation in plants is catalyzed by α-glucan water dikinase 1 (GWD1, EC 2.7.9.4) forming glycosyl-6-phosphate esters (Ritte et al., 2006), and the enzyme phosphoglucan water dikinase GWD3/PWD (EC 2.7.9.5), catalyzing the formation of glycosyl-3-phosphate esters in the starch (Baunsgaard et al., 2005; Kötting et al., 2005). Both enzymes use ATP as the phosphate donor. Gene suppression in potato (Lorberth et al., 1998) demonstrated that phosphorylation of starch by GWD1 stimulates starch degradation, supposedly by a starch granule amorphisising mechanism (Blennow and Engelsen, 2010). A similar effect was observed when GWD1 was used to phosphorylate starch *in vitro* (Ritte et al., 2002). However, other physiological effects of natural starch phosphorylation, such as a role in starch biosynthesis cannot be ruled out (Shaik et al., 2014; Skeffington et al., 2014). The use of GWD1 *in vivo* has been described in several patents including overexpression in wheat (Schewe et al., 2002) and corn (Lanahan and Basu, 2005) leading to increased viscosity of the starch paste. Hence hyperphosphorylation of starch *in planta* can provide an amorphisising and stabilizing modification. Despite the effects of GWD1 expression on starch degradation, overexpression of GWD1 in barley did not affect yield (Carciofi et al., 2011). To this date there are no reports on the use of GWD1 GWD3/PWD or any other starch phosphorylating enzymes for large scale *in vitro* fermentation, and therefore *in vivo* expression of such enzymes in the starch crops represents the main current biotechnological alternative to chemical phospho-esterification of starch. Modulating expression of GWD1 or GWD3/PWD is an obvious strategy to increase starch phosphate in crops. However, changing the expression of other enzymes in plant starch metabolism may also generate changes in starch phosphorylation. For example, increasing the lengths of amylopectin chains increases starch phosphorylation up to threefold (Schwall et al., 2000; Blennow et al., 2005).

## Hybrid Starch

Hybrid polysaccharides (HP) are defined as having covalent bonds between two or more different natural (poly)-saccharide systems. It is possible to engineer such HP by enzymatic polymerization *in vitro* by the action of different types of enzymes and their respective substrates, a technique which has been referred to as “enzymatic polymerization to unnatural HP” (Ohmae et al., 2007). Hybrid starches would be such unnatural HP, in which one of the components is amylose and/or amylopectin. Attempts have been made to engineer hybrid starches in plants using bacterial enzymes involved in the biosynthesis of extracellular polysaccharides (EPS) including α-glucans that are synthesized by sucrases. These include dextran, an α(1 → 6)-glucan synthesized by dextransucrase, mutan, an α(1 → 3)-glucan synthesized by mutansucrase, or alternan, an alternating α(1 → 3, 1 → 6)-glucan synthesized by alternansucrase. Expression of alternansucrase from the bacterium *Leuconostoc mesenteroides* in the amyloplasts of developing potato tubers resulted in lines with formation of small amounts of alternan (0.3–1.2 mg g^–1^ FW) (Kok-Jacon et al., 2007). A similar approach employing expression of dextransucrase from *L. mesenteroides* resulted in potato tubers containing between 1.0 and 1.7 mg g^–1^ FW dextran (Kok-Jacon et al., 2005). Expression of mutansucrase from *Streptococcus downei*, also in the potato amyloplast, resulted in formation of mutan inside the amyloplasts. All of these approaches resulted in irregular starch granule structures indicating modified starch types. However, none of these studies verified the presence of true novel hybrid covalently linked polysaccharides. It is possible that dextransucrase uses a mechanism where polymerization is primed by sucrose and the dextran polymer remains covalently bound to the enzyme during further polymerization (Robyt et al., 2008) and that such a mechanism prevents priming of dextran biosynthesis on amylose or amylopectin acceptor molecules. Fructans represent another type of potential polysaccharide for hybrid starch production. Levan, a common fructan, can be synthesized by levansucrases (GH68, EC 2.4.1.10) and *in vitro*, the *Bacillus subtilis* Levansucrase can catalyze fructosyl transfer to starch (Gerrits, 2000). Expression of this enzyme in potato tuber amyloplasts or tobacco chloroplasts resulted in the accumulation of fructan, however, again, no proof of covalent bonds between native starch and transgenic fructan was reported (Gerrits et al., 2001).

## Yield Penalty and *in planta* Starch Bioengineering

Plants generally demonstrate a tendency to display negative pleiotropic effects in response to transgenic events, which changes their starch biosynthesis (Kok-Jacon et al., 2003; Blennow et al., 2013). Strategies which modify starch biosynthesis producing higher or lower amylose levels usually results in moderate to large yield penalties (Yano et al., 1985; Oscarsson et al., 1998). Transgenic knock-down of SBE activity in barley gave a yield penalty of approximately 20% less grain weight per plant (Carciofi et al., 2012a). Similarly, storage starch composition and granule morphology is likely to have been shaped through evolution for optimal energy and biomass remobilization; therefore *in planta* starch modification in cereal grains can jeopardize germination efficiency and seedling establishment (Shaik et al., 2014). In other approaches, starch bioengineering in crops has been achieved without an apparent yield loss or other deleterious effects. Sweet potato expressing a hyperthermophilic α-amylase used for post-harvest starch hydrolysis was reported not to show any deviation from non-transgenic lines with respect to growth and yield, when grown under greenhouse conditions (Santa-Maria et al., 2011). In *Sorghum bicolor* allelic variation of a gene encoding the starch debranching enzyme pullulanase was shown to be a potential target for breeding toward higher starch digestibility without deleterious pleiotropic effects (Gilding et al., 2013).

## Conclusions and Perspectives

In this review we have compared the use of starch modifying enzymes produced by GM microorganisms with the expression of these enzymes directly in crops. In summary we find that *in planta* starch bioengineering by expression of starch modifying enzymes directly in crop storage organs faces a number of challenges that need to be addressed further. In particular, starch bioengineering may sometimes be associated with significant yield loss, e.g., by pleiotropic effects of the transgenic enzyme or due to effects of the modified starch structure on the ability of the starch storage organ to re-mobilize the energy that is stored in the starch. Only a few studies have been carried through to agronomic field trials. The physiological conditions in amyloplasts of crop starch organs may not be optimal for starch modifying enzymes of non-plant origin, and in several studies only very small amounts of the desired product is formed. However, the method looks promising for situations where the transgenic enzymes remain inactive during crop development, so that the above mentioned deleterious effects are avoided. For example crops expressing thermophilic hydrolytic enzymes, which are activated by heat, have been shown to reduce production costs and energy and water usage of grain processing. Other methods of “post-harvest” activation of transgenic enzymes in crops could be explored. In other situations there may not be a biotechnological alternative to transgenic enzyme expression directly in developing crop organs. For example starch kinases have been used to increase starch phosphate content in cereal grains and in potatoes, whereas there are currently no reports that a similar modification can be made during post-harvest starch processing by adding enzymes produced by GM microorganism.

### Conflict of Interest Statement

The Editor Giuseppe Dionisio declares that, despite being affiliated to the same institution as the authors Kim H. Hebelstrup and Domenico Sagnelli, the review process was handled objectively and no conflict of interest exists. The authors declare that the research was conducted in the absence of any commercial or financial relationships that could be construed as a potential conflict of interest.
